
JOURNAL INFORMATION
==============================
NLM Title Abbreviation: South Med Rec
NLM Journal ID: 0027317

The Southern Medical Record

Publisher: Biomedical Journal Digitization Project

ARTICLE INFORMATION
==============================
PMCID: PMC9094805
PMID: 36024127
Article version: 1
Subjects: Editorial

Dr. John B. Hamilton Resigns, &c.

Print publication date: 1889 Apr
Volume: 19
Issue: 4
First page: 159
Last page: 159
License: This work is free of known copyright restrictions. For more information, please see PMC Back Issue Digitization.
License URL: https://creativecommons.org/publicdomain/mark/1.0/

==============================

Dr. John B. Hamilton resigns his connection with the *Journal* of the American Medical Association, having resumed his life work in the Marine Hospital Service. The *Journal* of the Association will now be taken charge of by the Committee on General Management.

### SPECIAL NOTES.

**"Coca"** has maintained its reputation as a powerful nerve stimulant, being used with good results in nervous debility, opium and alcohol habit, etc. The highly variable character of the commercial drug makes it uncertain, however. Robinson's Wine Coca (see ads. p. 3) we believe to be a uniformly active article, it being prepared from assayed leaves, the per centage of Cocoaine being always determined by careful assay.

**Chloranodyne.** We have found great satisfaction in the use of Chloranodyne as prepared by Parke, Davis & Co. It is an anti emetic, and hence will often be borne by the stomach when morphine alone, or other anodyne remedies cannot be tolerated. It contains a minute quantity of morphia, well suited to delicate females or children. We have found that in a dose of 15 to 20 drops an eighth of a grain of morphia may be added with fine effect, the morphia being retained in almost every case when combined with the chloranodyne, even in cases of extreme gastric irritation.

**Reed & Carnrick.**—This well known and reliable House are getting out some most excellent preparations. Be sure to examine their advertisement in this number of our Journal relating to *Liquid Peptonoids Pancrobilin* and *Phospho-Caffein Comp.* The first has the remarkable property of dissolving diphtheretic deposit. So the other two preparations have very important properties.

**Bromidia, Papine and Iodia,** as prepared by Battle & Co., St. Louis, are all valuable preparations. Examine the advertisement of this House as found in this Journal.
